# Tomato fruits: a good target for iodine biofortification

**DOI:** 10.3389/fpls.2013.00205

**Published:** 2013-06-27

**Authors:** Claudia Kiferle, Silvia Gonzali, Harmen T. Holwerda, Rodrigo Real Ibaceta, Pierdomenico Perata

**Affiliations:** ^1^PlantLab, Institute of Life Sciences, Scuola Superiore Sant’AnnaPisa, Italy; ^2^SQM Europe N.V.Antwerpen, Belgium; ^3^SQM Industrial S.A.Santiago, Chile

**Keywords:** biofortification, iodine, iodine deficiency, potassium iodate, potassium iodide, *Solanum lycopersicum *L., tomato

## Abstract

Iodine is a trace element that is fundamental for human health: its deficiency affects about two billion people worldwide. Fruits and vegetables are usually poor sources of iodine; however, plants can accumulate iodine if it is either present or exogenously administered to the soil. The biofortification of crops with iodine has therefore been proposed as a strategy for improving human nutrition. A greenhouse pot experiment was carried out to evaluate the possibility of biofortifying tomato fruits with iodine. Increasing concentrations of iodine supplied as KI or KIO_3_ were administered to plants as root treatments and the iodine accumulation in fruits was measured. The influences of the soil organic matter content or the nitrate level in the nutritive solution were analyzed. Finally, yield and qualitative properties of the biofortified tomatoes were considered, as well as the possible influence of fruit storage and processing on the iodine content. Results showed that the use of both the iodized salts induced a significant increase in the fruit’s iodine content in doses that did not affect plant growth and development. The final levels ranged from a few mg up to 10 mg iodine kg ^-^^1^ fruit fresh weight and are more than adequate for a biofortification program, since 150 μg iodine per day is the recommended dietary allowance for adults. In general, the iodine treatments scarcely affected fruit appearance and quality, even with the highest concentrations applied. In contrast, the use of KI in plants fertilized with low doses of nitrate induced moderate phytotoxicity symptoms. Organic matter-rich soils improved the plant’s health and production, with only mild reductions in iodine stored in the fruits. Finally, a short period of storage at room temperature or a 30-min boiling treatment did not reduce the iodine content in the fruits, if the peel was maintained. All these results suggest that tomato is a particularly suitable crop for iodine biofortification programs.

## INTRODUCTION

The health and well-being of a population are significantly influenced by their nutritional status. A healthy and well-balanced diet, with a variety of high-quality foods ensuring the right proportions of different types of nutrients, is important both in the prevention and in the treatment of several diseases. Not only do daily calorie requirements need to be carefully met, but also the consumption of a number of specific elements, the lack of which may promote or lead to serious pathologies, needs to be guaranteed in order to prevent nutritional deficiencies.

Iodine (I) is a trace element used in the synthesis of thyroid hormones (Arthur and Beckett, 1999). It is naturally present in fish, eggs, meat, dairy products, and, to a lesser extent, in grains, fruits, and vegetables. For an adult the recommended daily allowance (RDA) for iodine is 150 μg (Institute of Medicine, Food and Nutrition Board, 2001), a very minute quantity. Nevertheless, its deficiency is one of the most serious public health issues worldwide and nearly one-third of the human population still has an insufficient iodine intake (Andersson et al., 2012). This is due to the fact that iodine deficiency is largely related to the environment. In many regions of the world, mountainous areas and flood plains in particular, soils contain very low amounts of iodine, which negatively affects the iodine content of crops, thus increasing the risk of iodine deficiency among people who consume foods primarily produced there.

Inadequate iodine intake impairs the thyroid function, with the onset of a wide spectrum of disorders negatively affecting growth and development at various levels. All age groups can be susceptible, and in cases of severe deficiency, damage to the fetus, perinatal and infant mortalities, endemic goitre, irreversible mental retardation and brain damage can occur (Delange, 2000; Zimmermann et al., 2008). Such problems are widespread in all the world’s least industrialized nations, with South Asia and sub-Saharan Africa particularly affected (Zimmermann, 2009). However, even in developed countries some groups of people remain at risk, especially children and pregnant women, resulting in minor cognitive and neuropsychological deficits (Haddow et al., 1999).

The main strategy for controlling and preventing iodine deficiency is the universal fortification of salt with iodine (Andersson et al., 2010). “Universal” is the key word in this strategy because it highlights that all the salt consumed by the population should be iodized, including salt used in food processing and for animal feed. This strategy has been implemented by many countries over the past few decades and has dramatically reduced the prevalence of iodine deficiency worldwide (Zimmermann, 2009; Andersson et al., 2010). However, a boost to the consumption of iodized salt is becoming increasingly untenable, as it conflicts with other important public health objectives, such as the prevention of cardiovascular diseases. Other strategies have been adopted, including the addition of iodine to oils, bakery products, or even to drinking water, but none of these alternatives has proved effective by itself as a means of prevention.

The biofortification of edible crops, based on the production of micronutrient-rich plants destined for human consumption, is a more recent alternative approach to controlling mineral malnutrition, especially in poor countries (Nestel et al., 2006). Biofortified crops may contain higher amounts of specific micronutrients due to their improved ability to take up and accumulate them or through a lower content of antinutrient compounds. These crops can be obtained by selecting superior genotypes through the use of traditional breeding or modern biotechnology. In alternative, improved agronomic approaches can be developed and applied (White and Broadley, 2009).

Although necessary for humans and animals, the importance of iodine for higher plants and a possible role in their metabolism have not yet been demonstrated. Usually fruits and vegetables are poor sources of iodine, although with large variations due to the differences in the iodine content of soils. However, several studies indicate that plants can accumulate iodine, and there is generally a positive correlation between applications to the soil and the final accumulation in plants (Zhu et al., 2003; Dai et al., 2004; Blasco et al., 2008; Weng et al., 2008a). The iodine biofortification of crops might thus be a cost-effective strategy for increasing iodine levels in plant-derived food, and thus improve human nutrition.

Several methods of iodine plant enrichment have been proposed, but none of these can be considered as optimal and each species requires a careful and specific evaluation. Although the positive results obtained in trials carried out with some leafy vegetables (e.g., spinach, lettuce), particularly in hydroponic culture, have suggested that they are good candidates for iodine biofortification programs (Zhu et al., 2003; Dai et al., 2004; Blasco et al., 2008; Hong et al., 2008; Weng et al., 2008b; Voogt et al., 2010), the fortification of other kinds of cultivated plants appears more difficult. Cereals, in particular, seem to be less suitable for such approaches, due to the scarce iodine accumulation levels in the grains (Mackowiak and Grossl, 1999), which in turn may be due to an insufficient phloematic route for iodine and/or a high volatilization rate of iodine from the plant to the atmosphere (Redeker et al., 2000; Landini et al., 2012).

Tomato (*Solanum lycopersicum* L.) is one of the most widely grown and commercially important vegetable crops, with a worldwide cultivation covering more than four million hectares (FAOSTAT, 2011). It is cultivated as an annual crop in open fields and under greenhouse conditions for both fresh consumption and industrial processing. The nutraceutical properties of tomato are well-known and are mainly related to the antioxidant potential of its fruits, due to the presence of a mix of bio-molecules such as lycopene, ascorbic acid, polyphenols, potassium, folate, and α-tocopherol (Basu and Imrhan, 2007).

Recent studies have proposed tomato as a possible candidate for iodine biofortification programs (Landini et al., 2011). Both its widespread distribution and possible consumption as a fresh fruit make it a good target crop for a fortification study. Indeed, positive results in terms of effective iodine accumulation within the fruits, representing the edible part of the plant, have been achieved (Landini et al., 2011).

In the present study an iodine biofortification approach was attempted using a commercial variety of tomato grown in potting soil in a greenhouse. Various agronomic aspects that may or may not influence the availability of iodine for plant uptake were analyzed, for example the iodine source and dose, the type of soil, and the concentration of other nutrients. The final effects were also analyzed in terms of quantitative yield and qualitative properties of the biofortified tomatoes, as well as the possible influence of fruit storage and processing.

## MATERIALS AND METHODS

### PLANT MATERIAL AND GROWTH CONDITIONS

The tomato variety SUN7705 (Nunhems, Parma, ID 83660, USA) was used in all the experiments. Seeds were sown in soil (Hawita Flor, Vechta, Germany) in plastic plugs and in a growth chamber under the following conditions: 25°C temperature, 55% relative humidity, 80 μmol m^-^^2^ s^-^^1^ PAR (photosynthetically active radiation). From germination to transplanting, plants were watered once a week with a nutritive solution, whose composition was the same as that used in the pot cultivation (see later in this paragraph). About 40 days after germination, tomato plants were transplanted to 24 cm diameter plastic pots (volume = 8 dm^3^) containing a mixture of soil and pumice (70:30, by volume), and transferred to a glass greenhouse. Pumice was used in order to facilitate water drainage. The main characteristics of the soil were: clay 8.4%, silt 32.0%, sand 59.6%; C/N 8.5; organic matter 1.31%; and electrical conductivity 0.44 mS cm^-^^1^. Throughout the trial, day/night temperatures ranged from 25 to 31°C, and from 15 to 21°C, respectively. The composition of the nutritive solution, supplied to plants for 1 min three times per day, was: (in mM) N-NO_3_ 11; N-NH_4_ 0.5; P 1.2; K 7; Ca 4; Mg 0.94; Na 10; Cl 9.5; S-SO_4_ 2.16; and (in μM) Fe 45; B 23; Cu 1; Zn 5; Mn 10; Mo 1; EC 2.79 mS cm^-^^1^, and pH between 5.7 and 6.0. The moderate sodium and chloride content was due to the use of slightly saline irrigation water.

For pest management, a foliar application of copper was performed before transplanting to prevent tomato blight. Confidor (Bayer, Germany) was applied as a foliar application against aphids and white flies, once a week from transplanting to flowering. In addition, a systemic fungicide (Ridomil Gold^®^ EC, Novartis, NY, USA) was applied to the soil every 10 days from transplant to harvest.

Different experimental trials were performed, as later described. In all the trials, iodine treatment administrations were carried out, supplying iodine to pots as KI or KIO_3_, dissolved in a volume of 200 ml water per plant. KI or KIO_3_ concentrations ranged from 0 to 10 mM, depending on the type of experiment. Treatment applications were carried out weekly, starting from the development of the first branch of fruits.

### EXPERIMENT 1: EFFECT OF IODINE DOSE AND FORM ON IODINE UPTAKE AND ACCUMULATION

Tomato plants were grown in soil in a glass greenhouse, fertirrigated with a nutritive solution, as above described. Starting with the set of the first truss of fruits, plants were root-treated with KI or KIO_3_ once a week. Eight iodine administrations were performed.

Following a preliminary trial, with a very wide iodine dose–response curve (KI and KIO_3_ concentrations ranging from 0 to 60 mM), performed to find out the most suitable doses of iodine without phytotoxicity symptoms, KI was supplied in concentrations of 1, 2, and 5 mM, while KIO_3_ in concentrations of 0.5, 1, and 2 mM. Ten replicates for each experimental condition were carried out.

After the first four iodine administrations (total effective iodine supplied per plant: 0, 50.76, 101.52, 203.04, 507.6, and 1,015.2 mg I, corresponding, respectively, to 0, 0.5, 1, 2, 5 and 10 mM I applied as KI or KIO_3_), the iodine content was measured in fruits from both the first and the second trusses. Other four iodine treatments were then carried out and the iodine content was measured in fruits collected from the first truss and used for the qualitative analyses.

### EXPERIMENT 2: EFFECT OF SOIL ORGANIC MATTER ON IODINE UPTAKE AND ACCUMULATION

Plants were grown in pots in a glass greenhouse, fertirrigated with a nutritive solution, as above described, and divided into two groups, according to the organic matter content of the soil mixture used. Two different soil mixtures, characterized by a low and a high organic matter content, respectively, were used. The composition of the soil mixture with the low organic matter content (approximately 1% on a weight base) was the same described above (soil:pumice, 70:30 by volume). The soil mixture with the high organic matter content was obtained by mixing soil, commercial peat (Hawita Flor) and pumice (28:41:30, by volume), considering the main characteristics of the different substrates, that were, respectively: organic matter content: 1.31, 40, and 0% on a dry matter basis; apparent density (kg/L): 1.5, 0.5, and 0.85 on a dry matter basis; dry matter content: 90, 72, and 90%. In the mixture soil enriched with peat the final organic matter content was approximately 10% (determined on a weight base).

Starting from the development of the first branch of fruits, four weekly administrations of 10 mM KI or KIO_3_ (total effective iodine supplied per plant: 1,015.2 mg I) were performed in both the two groups of plants. Control plants, untreated with iodine, were also grown in the two types of soils. Ten replicates for each experimental condition were carried out.

Fruits were collected from the first fruit cluster at the end of the iodine treatments and analyzed for the iodine content. At the end of the trial, some plant growth parameters (fruit and shoot dry weight, fruit yield) were measured.

### EXPERIMENT 3: EFFECT OF THE NITRATE LEVEL OF THE NUTRITIVE SOLUTION ON IODINE UPTAKE AND ACCUMULATION

Plants were grown in soil in a glass greenhouse, as above described, and divided into three different groups, according to the nitrate level of the nutritive solution. Three different nutritive solutions, containing, respectively, a low (2 mM), medium (10 mM), and high (20 mM) nitrate content, were used. The medium nitrate nutritive solution had the following composition: (in mM) N-NO_3_ 10; P 1.2; K 8; Ca 6; Mg 1; Na 10; Cl 9.5; S-SO_4_ 4.97; and (in μM) Fe 56; B 23; Cu 1; Zn 5; Mn 11; Mo 1; EC 3.32 mS cm^-^^1^, and pH between 5.7 and 6.0. The low nitrate nutritive solution contained 2 mM N-NO_3_, while the high nitrate one 20 mM N-NO_3_. Furthermore, some adjustments were made to the low and high nitrate solutions to maintain comparable macronutrient levels. The low nitrate nutritive solution contained additional 7 mmol l^-^^1^ chloride, while in the high nitrate solution the sulfate content was reduced to 0.8 mM.

Starting from the development of the first branch of fruits, four weekly administrations of 10 mM KI or KIO_3_ (total effective iodine supplied per plant: 1,015.2 mg I) were performed in all the three groups of plants. Control plants, untreated with iodine, were also grown with each of the three different nutritive solutions. Ten replicates for each experimental condition were carried out.

Fruits were collected from the first fruit cluster at the end of the iodine treatments and analyzed for the iodine content. At the end of the trial, some plant growth parameters (fruit and shoot dry weight, fruit yield) were measured.

### EXPERIMENT 4: EFFECT OF SHELF-LIFE AND COOKING ON THE IODINE ACCUMULATED IN TOMATO FRUITS

For this experiment, fruits collected from plants of the Experiment 1, treated for 4 weeks with 5 mM KI (total effective iodine supplied per plant: 507.6 mg I) were used. Both turning red and red fruits were chosen.

The shelf-life experiment was performed by storing the turning red fruits under light at room temperature without any treatment for the following 2 weeks after harvest. The analyses of the iodine content on the stored fruits were carried out 1 and 2 weeks after harvest.

The cooking experiment was performed by boiling red tomato fruits for 30 min in deionized water. Processed fruits were divided into two groups, and boiled, with or without the external peel, respectively.

### EXPERIMENT 5: EFFECT OF IODINE ON FRUIT QUALITY

Fruits collected from plants of the Experiment 1 treated for 8 weeks with KI or KIO_3_ were used. Both quantitative measures (fruit yield) and qualitative analyses (color, sugar content, total antioxidant power) were carried out.

### QUALITATIVE AND QUANTITATIVE ANALYSES OF FRUITS

For the analysis of the iodine content, fruits were harvested waiting at least 1 week after the last iodine treatment. Iodine as I was analyzed by inductively coupled plasma mass Spectrometry (ICP-MS), as previously described (Landini et al., 2011).

For the evaluation of dry weight (DW), fruits and shoots were weighed separately immediately after harvest and then dried in a ventilated oven at 80°C. All the fruits collected from plants at the end of the experiments were weighed for the analysis of fruit yields. Experiments were not continued after the collection of fruits from the first two branches. The calculated yields therefore always refer to the fruits collected from these two trusses, already developed, and those still growing in the third truss.

In order to analyse sugar content, whole fresh fruits were homogenized in a blender. An aliquot of the homogenate was then centrifuged twice for 10 min at 5,000 rpm and some drops of the supernatant were used to determine total soluble solids with a Refractometer (RL3 PZO). The content of sugars was expressed as degrees Brix (°Brix).

The total antioxidant power of fruits was evaluated using the “ferric-reducing/antioxidant power” (FRAP) assay (Benzie and Strain, 1996). Immediately after harvest, each fruit was homogenized in a blender (0.5 g of the flesh extracted in 5 ml of pure methanol) and stored overnight at -20°C. Samples were then centrifuged for 8 min at 5.000 rpm and 100 μl of the supernatant were added to 900 μl of freshly prepared FRAP reagent [1 mM TPTZ + 2 mM FeCl_3_] and 2 ml of acetate buffer. Absorbance readings at 593 nm were taken after a reaction time of 4 min. The reagents used were: acetate buffer (0.25 M sodium acetate, pH 3.6), TPTZ (2,4,6-tripyridyl-2-triazine 0.01 M in methanol) and FeCl_3_ (0.01 M in sodium acetate). Standard solutions of known Fe^2^^+^ concentration (0–50-200-500-1000 μM) were prepared by adding (NH_4_)2Fe(SO_4_)_2_·6 H_2_O to the acetate buffer, and were used for the calibration.

### STATISTICAL ANALYSIS OF DATA

The experimental design adopted in the Experiments 1, 2, and 3 was completely randomized. The treatments (iodine source and organic matter in the Experiment 2; iodine source and nitrate level in the Experiment 3, respectively) were in factorial combination. Data were subjected to one-way and two-way analysis of variance (ANOVA; Statgraphics Centurion XV program), as described in the Figure legends, and the means were separated using the *F*-test (95% confidence level).

## RESULTS

### EXPERIMENT 1: EFFECT OF IODINE DOSE AND FORM ON IODINE UPTAKE AND ACCUMULATION

**FIGURE 1:**
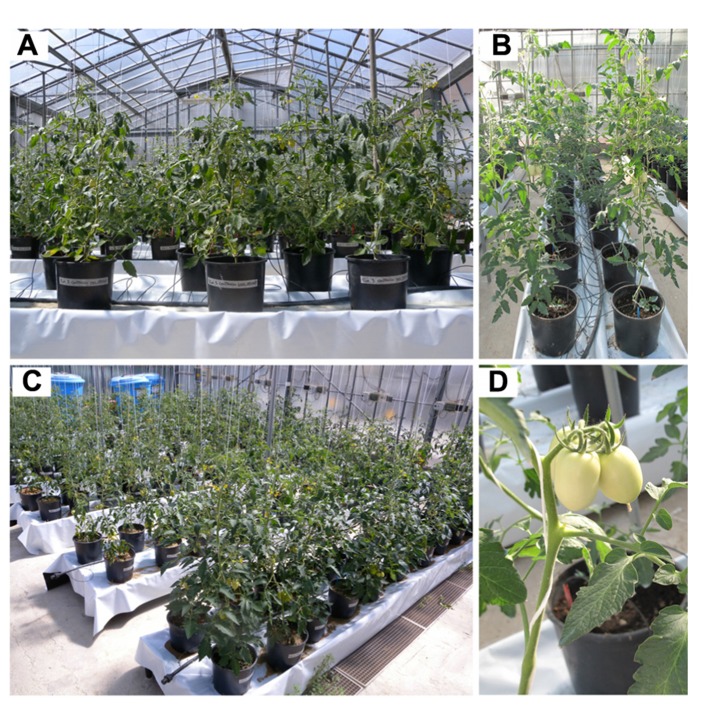
**Set-up of tomato plant greenhouse cultivation.** Plants were grown in pots **(A)** and organized in rows **(B)**, fertilized with a nutritive solution **(C)**. Iodine treatments started with the set of the first truss of fruits **(D)**.

As a starting point, tomato plants, grown with the experimental set-up previously described (**Figure 1**), were root-treated with KI or KIO_3_ concentrations ranging from 0 to 60 mM. Although clear damage was never observed in the fruits, plants started to show phytotoxicity symptoms (leaf chlorosis, epinasty, visible wilting) at iodine salt concentrations higher than 10 mM. Moreover, in the presence of 40–60 mM KI or KIO_3_, plant development and biomass accumulation were severely compromised, with undeniable consequences on the development of the fruits (data not shown).

**FIGURE 2:**
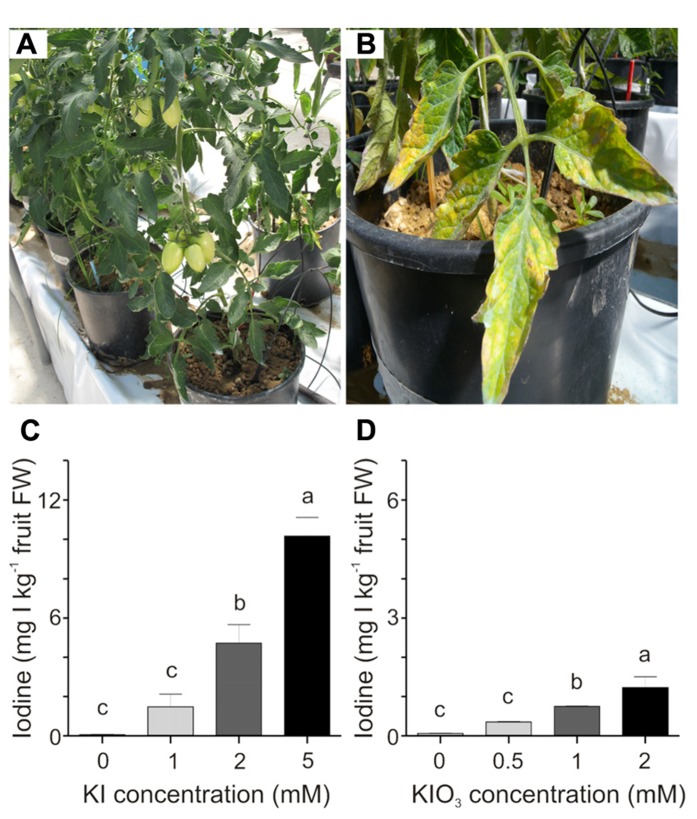
**Effect of iodine dose and form on iodine uptake and accumulation.** Healthy iodine-treated plants at the end of the experiment **(A)**. 5 mM KI-treated plants showed some phytotoxicity symptoms on the basal leaves **(B)**. Iodine uptake in fruits collected from the second truss of **(C)** KI- and **(D)** KIO_3_-treated plants, after four weekly iodine applications. Data were subjected to one-way analysis of variance (ANOVA) and the means were separated using the *F*-test (95% confidence level).

These preliminary results prompted us to focus on a narrower and lower range of iodine concentrations, to limit any phytotoxicity symptoms on the plants. KI was thus supplied in concentrations of 1, 2, and 5 mM, while KIO_3_ was used at lower concentrations, namely 0.5, 1, and 2 mM, since this salt showed greater phytotoxicity in the preliminary trial. In this experimental set-up, the plants were healthy at the end of the experiment (**Figure 2A**), with the exception of those treated with the highest KI concentration (5 mM) which showed some discoloration and necrotic areas, limited to the basal leaves (**Figure 2B**).

The trial was interrupted when the third truss of fruits was developing and the iodine content was measured in fruits from both the first and the second trusses. **Figures 2C,D** show the iodine content detected in fruits collected from the second branch at the mature green stage. A very regular trend in the increase in fruit iodine content with the increase in its soil administration can be observed. After four treatments with 1, 2, and 5 mM KI, fruits contained an average of 1.5, 4.7, and 10 mg I kg^-^^1^ fresh weight (FW), respectively (**Figure 2C**). A similar trend can be observed in fruits from the plants treated with potassium iodate, with fruits accumulating 0.3, 0.7, and 1.2 mg I kg^-^^1^ FW following four applications of 0.5, 1, and 2 mM KIO_3_, respectively (**Figure 2D**). Comparable results were obtained in fruits collected from the first truss (data not shown). In the analyses performed, the untreated control fruits showed a small amount of iodine (approximately 0.06 mg I kg^-^^1^ FW) due to the trace amounts of this element present in both the irrigation water (0.109 mg I l^-^^1^) and the soil used (0.084 mg I kg^-^^1^); (**Figures 2C,D**).

### EXPERIMENT 2: EFFECT OF THE SOIL ORGANIC MATTER ON IODINE UPTAKE AND ACCUMULATION

**FIGURE 3:**
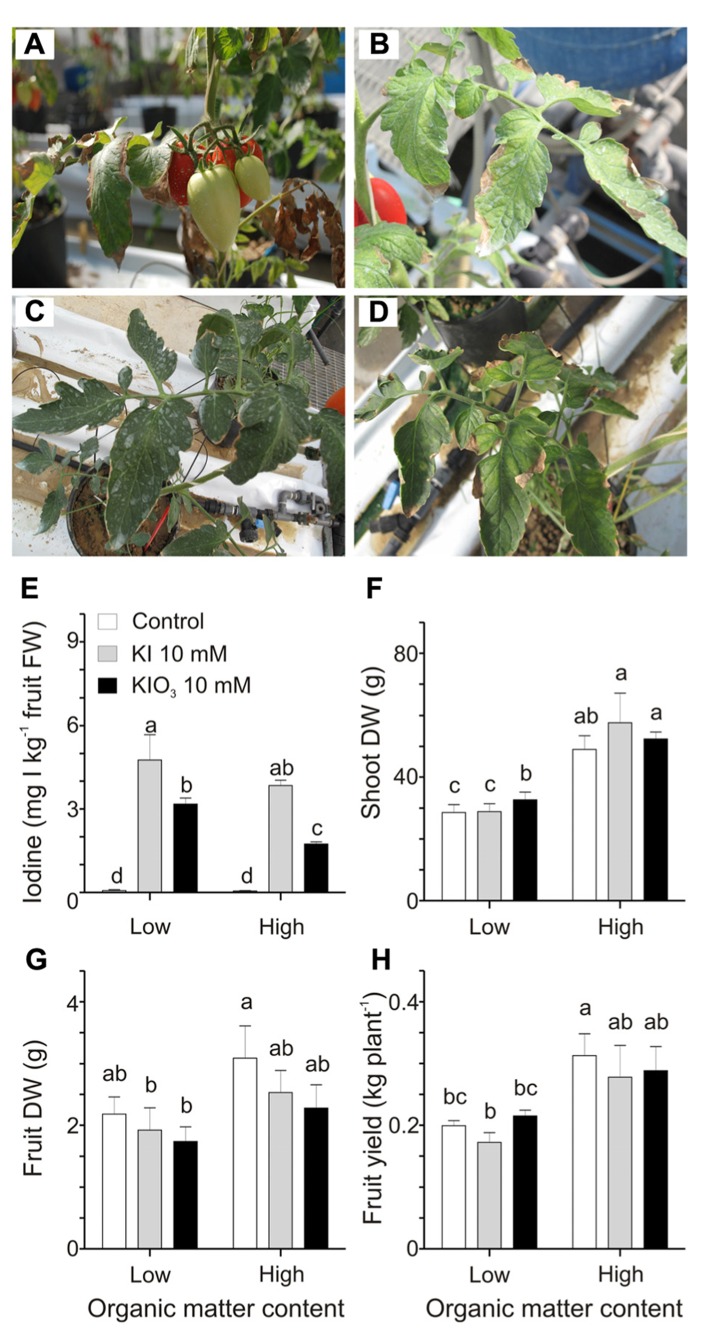
**Effect of the soil organic matter on iodine uptake and accumulation.** Details of leaves and fruits from plants grown in a soil with 1% organic matter treated with 10 mM KI **(A)** or 10 mM KIO_3_ **(B)**, and from plants grown in soil with 10% organic matter treated with 10 mM KI **(C)** or 10 mM KIO_3_ **(D)**. Iodine levels in fruits **(E)**, shoot dry weight (DW) **(F)**, fruit DW **(G)**, and fruit yield **(H)** measured in plants grown in low and high organic matter soils and with or without a 10 mM KI or 10 mM KIO_3_ treatment. Data were subjected to one-way and two-way analysis of variance (ANOVA) and the means were separated using the F-test (95.0% confidence level). Significance of two-way analysis of variance (*P-value ≤ 0.05; ***P-value ≤ 0.001; n.s. = not significant): **(E)** organic matter content (a): *; iodine treatment (b): ***; a x b: n.s.. **(F)** organic matter content (a): ***; iodine treatment (b): n.s.; a x b: n.s.. **(G)** organic matter content (a): *; iodine treatment (b): n.s.; a x b: n.s.. **(H)** organic matter content (a): ***; iodine treatment (b): n.s.; a x b: n.s..

In this second experiment, plants, grown into low or high organic matter soils, were treated with 10 mM KI or KIO_3_, a concentration of iodine higher than those used in the previous trial, chosen to better quantify the possible negative effects of the organic matter on the iodine uptake. Over the 4 weeks of treatments, mild phytotoxicity symptoms appeared on the plants, depending on the form of iodine administered as well as the soil organic matter content. The most affected plants were those grown in the lower organic matter soil and treated with KI. Leaves of this group of plants presented some discolorations and necrotic areas (**Figure 3A**). Similar phytotoxic effects, though less severe, were observed on plants treated with KIO_3_ (**Figure 3B**). In the high organic matter content soil all the plants appeared healthier (**Figures 3C,D**).

Fruits were collected from the first fruit cluster and analyzed for the iodine content (**Figure 3E**). The results obtained show that the increase in the organic matter reduced the iodine accumulation in KIO_3_- but not in KI-treated plants.

Some plant growth parameters were analyzed in order to better quantify the effects of the different types of soil in combination with the iodine treatments. Plants grown in organic matter-rich soils showed a strong increase in the dry-matter production of their vegetative organs (**Figure 3F**), which was, on average, 1.5-fold higher than that quantified in the low organic matter soil. This effect was observed irrespectively of the iodine treatment performed. No significant effects were detected in fruit dry weight (**Figure 3G**), while plant yield was positively affected by the organic matter, as, on average, plants grown in the organic matter-enriched soil showed a fruit production 1.5-fold higher than those grown in the organic matter poor soil, but, again, this was observed irrespectively of the iodine treatment performed (**Figure 3H**).

### EXPERIMENT 3: EFFECT OF THE NITRATE LEVEL OF THE NUTRITIVE SOLUTION ON IODINE UPTAKE AND ACCUMULATION

**FIGURE 4:**
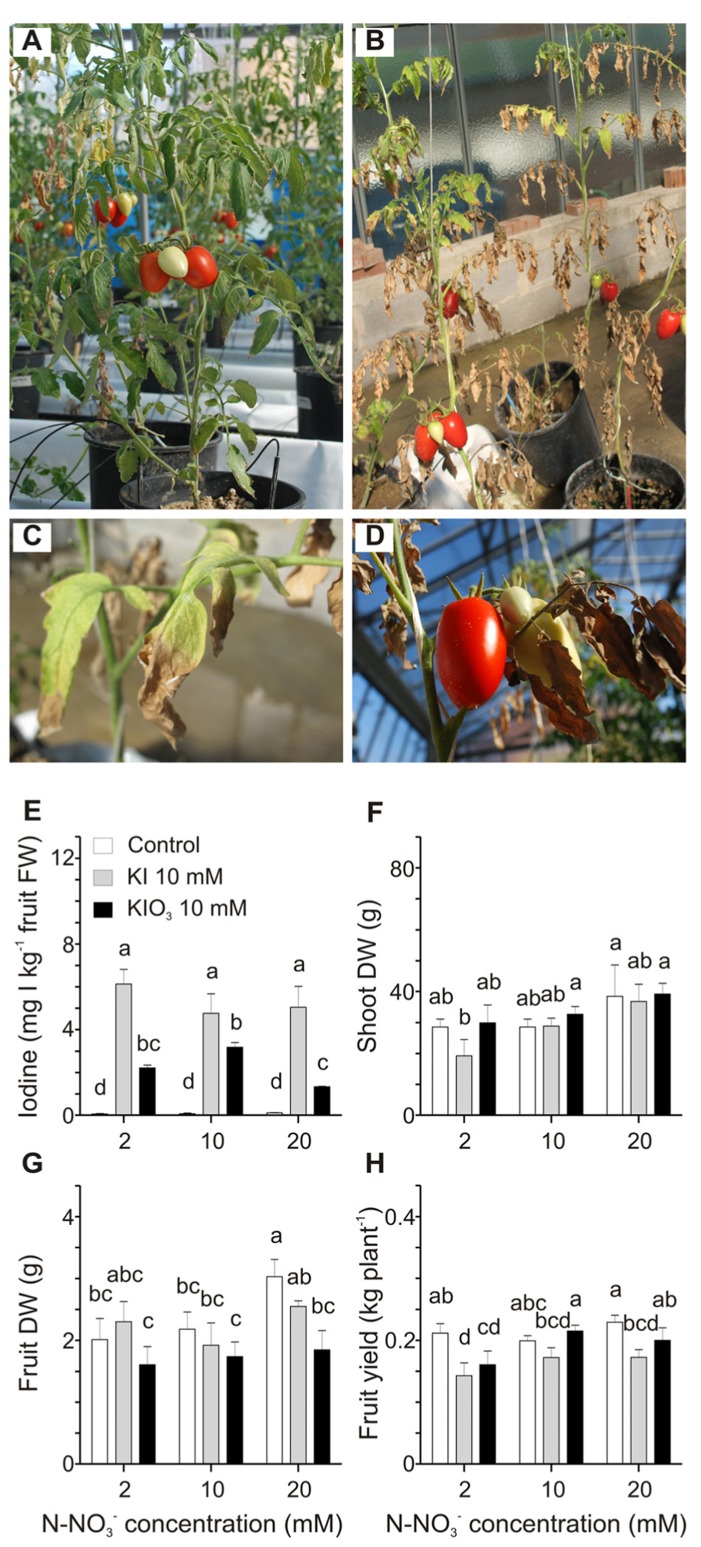
**Effect of the nitrate level of the nutritive solution on iodine uptake and accumulation.** Plants fertilized with 2 mM nitrate without iodine treatments **(A)**, or treated with 10 mM KI **(B)** are shown. Details of leaves from the upper branches **(C)** and fruits **(D)** from plants fertilized with 2 mM nitrate and treated with 10 mM KI. Iodine levels in fruits **(E)**, shoot dry weight (DW) **(F)**, fruit DW **(G)**, and fruit yield **(H)** measured in plants fertilized with 2, 10 or 20 mM nitrate level and with or without 10 mM KI or 10 mM KIO_3_ treatments. Data were subjected to one-way and two-way analysis of variance (ANOVA) and the means were separated using the F-test (95.0% confidence level). Significance of two-way analysis of variance (*P-value ≤ 0.05; ***P-value ≤ 0.001; n.s. = not significant): **(E)** nitrate concentration (a): n.s.; iodine treatment (b): ***; a x b: n.s.. **(F)** nitrate concentration (a): *; iodine treatment (*b*): n.s.; a x b: n.s.. **(G)** nitrate concentration (a): n.s.; iodine treatment (b): *; a x b: n.s.. **(H)** nitrate concentration (a): n.s.; iodine treatment (b): *; a x b: n.s..

The possible interaction between iodine and nitrate contained in the nutritive solution in terms of iodine availability and uptake by tomato plants was examined. Three different nitrate doses (2, 10, and 20 mM) were used to fertilize plants, which were also treated with 10 mM KI or KIO_3_. Strong phytotoxicity symptoms on plants treated with KI and grown at the minimal nitrate level (2 mM) were observed (**Figure 4B**). These plants were strongly reduced in size and biomass production in comparison with plants fertilized with 2 mM nitrate but not treated with KI (**Figure 4A**), and, at the end of the trial, their basal leaves were completely burnt (**Figure 4B**). Leaves of the upper branches still showed chlorosis, necrotic areas, curling of the edges and a reduction in size (**Figures 4B,C**), whereas fruit appearance did not seem to be affected (**Figure 4D**). Similar phytotoxic effects, though less severe, were observed in plants grown at 2 mM nitrate dose and treated with KIO_3 _ (data not shown). On the other hand, iodine-treated plants fertilized with 10 and 20 mM nitrate did not show significant alterations in their growth, apart from some chlorotic and necrotic areas on the basal leaves of plants treated with KI. Control plants, not treated with iodine, also showed a slight chlorosis when fertigated with 2 mM nitrate (**Figure 4A**). The final amount of iodine in fruits collected from plants treated with the same iodine salt and increasing doses of nitrate was comparable (**Figure 4E**). Only fruits from 10 mM KIO_3_-treated plants fertilized with 20 mM nitrate showed a small but significant reduction in the iodine content (**Figure 4E**).

Plant dry weight and yield were measured. A significant reduction in shoot DW was observed only in plants treated with 2 mM nitrate and 10 mM KI (**Figure 4F**), as a likely consequence of the strong iodine phytotoxicity under these conditions (**Figure 4B**). As far as fruit DW is concerned, no significant differences were detected in iodine-treated plants, whereas in control plants, not treated with iodine, a small trend toward a slight increase can be observed comparing, respectively, the 10 and 20 mM nitrate levels (**Figure 4G**). Finally the level of nitrate fertilization did not significantly affect the fruit yield in control plants, whereas the KI treatment reduced fruit yield in all the plants and in particular in those grown at the lowest nitrate concentration (**Figure 4H**), probably due to the phytotoxic effects described above. On the contrary, fruit yield in KIO_3_-treated plants slightly increased with the increase in the nitrate level (**Figure 4H**).

### EXPERIMENT 4: EFFECT OF SHELF-LIFE AND COOKING ON THE IODINE ACCUMULATED IN TOMATO FRUITS

**FIGURE 5:**
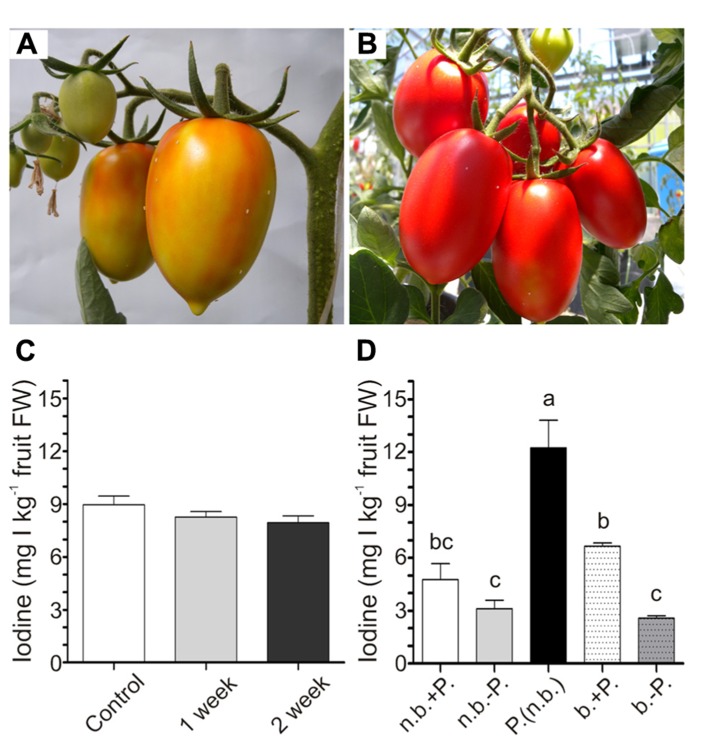
**Effects of shelf-life and cooking on iodine content of fruits.** Fruits turning red at harvest for the shelf-life experiment **(A)**. Red fruits at harvest for the boiling experiment **(B)**. Iodine content in 5 mM KI-treated fruits at harvest, and after one or 2 weeks of storage at room temperature **(C)**. Iodine content in 5 mM KI-treated fruits at harvest not boiled (n.b.) or boiled (b.) with (+P.) or without (-P.) peel, or in fruit skin not boiled [P. (n.b.)] **(D)**. Data were subjected to one-way analysis of variance (ANOVA) and the means were separated using the *F*-test (95% confidence level).

To evaluate the possible effect of storage on the level of iodine accumulated in tomatoes, fruits were collected from 5mM KI-treated plants at the breaker stage (**Figure 5A**). The shelf-life experiment was performed by storing the fruits under light at room temperature without any further treatment for the following 2 weeks, during which fruit ripening continued. The iodine content remained constant in the fruits over time (**Figure 5C**), showing that 2 weeks of storage did not alter their value of biofortified fruits.

To evaluate the possibility of transforming the iodine-enriched tomatoes into processed food, a cooking experiment was performed by boiling red ripened fruits (**Figure 5B**) for 30 min. Both raw and processed fruits were divided into two groups, maintaining or removing the external peel. Iodine was finally measured in intact and peeled fruits and also in the fruit skin. Boiling did not alter the amount of iodine present in fruits, and, irrespectively of the treatment, the content of iodine in fruits without peel was lower than that measured in the same intact fruits (**Figure 5D**). Indeed, the peel alone contained very high levels of iodine (**Figure 5D**).

### EXPERIMENT 5: EFFECT OF IODINE CONTAINED IN FRUITS ON THEIR QUALITY

**FIGURE 6:**
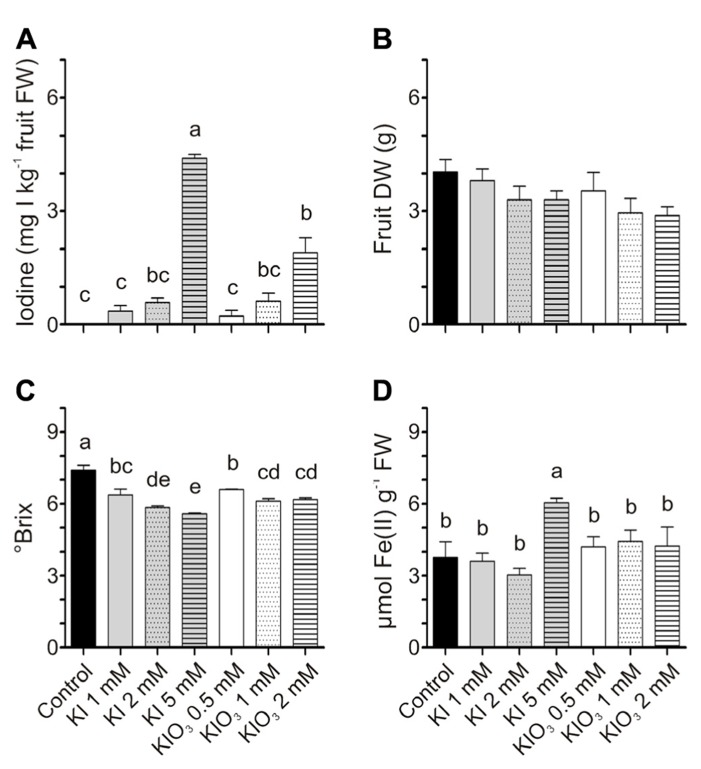
**Effect of iodine on fruit quality.** Iodine levels **(A)**, dry weight (DW)** (B)**, sugar content **(C)**, and antioxidant capacity **(D)** measured in fruits collected at the red ripening stage from the first truss of plants treated eight times with 0–5 mM KI or 0–2 mM KIO_3_. Data were subjected to one-way analysis of variance (ANOVA) and the means were separated using the *F*-test (95% confidence level).

For the qualitative analyses of fruits, tomatoes were harvested at the red stage of ripening. In this experiment, iodine treatments were thus prolonged, and fruits were collected after eight iodine applications. Iodine accumulated in these fruits (**Figure 6A**) with a trend similar to that previously observed after four administrations (**Figures 2C,D**). The values of the iodine content, with the exception of a few samples, were also comparable. Fruits from KI-treated plants accumulated approximately 0.3–4.5 mg I kg^-^^1^ FW following treatments with 1–5 mM KI respectively, while fruits from 0.5 to 2 mM KIO_3_-treated plants ranged from 0.2 to 1.9 mg I kg^-^^1^ FW (**Figure 6A**). The DW of fruits was not significantly different (**Figure 6B**).

Fruit quality was evaluated in terms of sugar content and antioxidant power. Treatments with KI and KIO_3_ mildly reduced the fruit sugar content, as the °Brix progressively decreased, slightly but significantly, with the increase in potassium iodide or iodate concentrations (**Figure 6C**). On the contrary, no significant differences were detected in the ferric-reducing ability of tomatoes, with the exception of the value measured in fruits from 5 mM KI-treated plants, which was significantly higher than the control (**Figure 6D**). These fruits were those that accumulated the highest level of iodine (**Figure 6A**).

## DISCUSSION

In accordance with the previous positive results obtained in hydroponic-grown tomato plants (Landini et al., 2011), the experimental trials presented here clearly indicate that even in soil-grown plants iodine can be accumulated in fruits at very high levels. Both KI and KIO_3_ administered to the soil can be efficiently taken up by the roots and the iodine amounts detected in fruits may be adequate for a biofortification program without using iodine doses that are toxic to the plant. On the whole, the most suitable iodine concentrations for a satisfactory biofortification of fruits were the lowest tested in the Experiment 1, corresponding to 0.5–1 mM, of both iodide and iodate (**Figure 2**). In our growth conditions, these doses corresponded to 12.7 and 25.4 mg I per single treatment application, respectively, and with a volume of about 8 dm^3^ soil per pot, as in our case, to approximately 1.6–3.2 mg I dm^-^^3^ soil. Therefore, a weekly fertigation with these doses of KI or KIO_3_, starting from the first fruit stage of development, could lead to a final accumulation of iodine in the fruit that would be suitable for a biofortification program.

We started the applications of iodine at the onset of the first fruit cluster. During tomato growth, most of the fruit weight is accumulated by the mature green stage (Ho and Hewitt, 1986; Srivastava and Handa, 2005), and there is recent evidence regarding the important role of the xylematic system in providing water to tomato trusses (Windt et al., 2009). It is therefore reasonable to assume that iodine can be more easily translocated during the early fast growth of the fruits and that the xylematic system is the main route for iodine translocation within the plant, if iodine is administered to the soil.

The iodine status of a soil is a combination of the supply of iodine and the soil’s ability to retain it. It is well-known that one of the most important components for the sorption of iodine in soils is the organic matter (Whitehead, 1974, 1978; Gerzabek et al., 1999; Yamaguchi et al., 2010), which can thus potentially affect the mobility of this element in the soil solution and its availability for plant uptake (Sheppard and Thibault, 1992; Hu et al., 2005; Dai et al., 2009). Results obtained in our Experiment 2 indicate that tomato plants grown in a high organic matter soil accumulated less iodine within the fruits if treated with KIO_3_ (**Figure 3E**), thus confirming the possible negative role of the organic matter on the mobility of iodine and also indicating that iodate could be retained stronger than KI by the organic matter fraction of the soil. Due to its direct and indirect effects on the availability of nutrients, organic matter can also interfere with plant development and productivity (Bauer and Black, 1994; Martin-Rueda et al., 2007; Rigane and Medhioub, 2011). In our trial, the soil with high organic matter content, irrespectively of the iodine treatment applications, positively affected the plant growth and productivity (**Figures 3F,H**). In addition, the mild phytotoxicity symptoms, observed almost exclusively on the KI-treated plants, were less severe in the presence of high organic matter in the soil (**Figures 3A,B**). Therefore, in order to select a soil type suitable for iodine biofortification programs, a careful evaluation of all these factors is required.

Evaluating any interactions between iodine and nitrogen (N) is crucial in order to develop optimal agro-techniques for tomato biofortification with iodine. Fertilization of the soil with N can influence the concentration of some microelements in the soil solution, either increasing (e.g., Fe, Cu, Zn, Mn) or reducing (e.g., B, Mo) their solubility (Rutkowska et al., 2009). Furthermore, possible inhibitory effects of nitrate on halide absorption by root plants, likely as a consequence of competition during plant uptake, have been described (Roorda van Eysinga and Spaans, 1985). In our Experiment 3, iodine levels accumulated in tomatoes were generally not influenced by the nitrate dose used in the fertilization of the plants, with only a minor negative effect of high nitrates in fruits from KIO_3_-treated plants (**Figure 4E**). However, nitrogen deficiency represented a stressful condition for plant growth and development and KI phytotoxicity symptoms were much more evident on plants grown at the minimal nitrate level (2 mM). Although we cannot rule out that the moderate salinity of the water used for the fertirrigation of the plants increased these effects, such symptoms were not found in plants grown in the same conditions without iodine applications (**Figure 4A**). Furthermore, nitrogen is one of the main nutrients required for plant growth and can also affect plant vigor and fruit quality (Shaahan et al., 1999; Tei et al., 2002). N supply is positively correlated with tomato yields (Guidi et al., 1998; Le Bot et al., 2001; Bernard et al., 2009). We did not detect significant effects on fruit yield production as a result of the nitrate concentration in the nutritive solution (**Figure 4H**). However, if our plants had been cultivated until a higher number of fruit trusses had been formed, there might have been a stronger effect of the low nitrogen supply on fruit yield. Our trials thus indicate that the standard nitrate concentrations (about 10 mM) that are used in tomato cultivation should not negatively affect iodine uptake and accumulation (**Figure 4E**), while the deficiency of nitrogen could have a negative synergistic effect with the phytotoxicity of iodide (Herrett et al., 1962) on plant development and productivity (**Figure 4B**).

Tomatoes are either sold as fresh fruits and therefore consumed after a certain period of storage, or they are processed in order to produce pastes, sauces, or peeled products. The ability of tomato plants to volatilize iodine, described in other plant species (Redeker et al., 2000; Rhew et al., 2003; Itoh et al., 2009), is at present not known. The results obtained in our Experiment 4 indicate that iodine accumulated in tomato fruits is persistent after harvest (**Figure 5C**). A short shelf-life should thus not reduce the biological value of the iodine-rich fresh tomatoes. However, many other factors can affect post-harvest storage of fruits (low temperatures, atmosphere, humidity, packaging), and are therefore worthy of analysis.

We also found that removing the peel from tomato fruits led to a heavy reduction in their iodine content, as the peel appeared to be very rich in this element (**Figure 5D**). On the other hand, boiling of the fruits did not further reduce their iodine content (**Figure 5D**). Therefore, in fruits for industrial processing or simply for cooking, the peel should be maintained in order to preserve a high iodine concentration. Of course, we only measured iodine before and after a single boiling process. We cannot exclude that other cooking methods or cooking at higher temperatures might lead to higher iodine losses.

As a whole, tomato fruits resulted in being able to accumulate high amounts of iodine. Not even when plants were treated with iodine levels exerting strong phytotoxic effects on the vegetative organs, did the fruits appear to be affected, probably due to the lower levels of the element accumulating in fruits compared to in the leaves and stems (Landini et al., 2011). However, a qualitative analysis is necessary to ascertain whether the presence of iodine in tomatoes affects their quality, and in the Experiment 5 we carried out a preliminary evaluation of it. Tomatoes are usually consumed at their stage of maximum organoleptic quality, which occurs when they reach the full red color, but before excessive softening. Our qualitative analyses were thus performed on fruits at the mature red stage of ripening. In concentrations of a few mg I kg^-^^1^ FW, such as those detected in our biofortified fruits, iodine did not alter the visual appearance of the fruits, which maintained their original size, shape, and color (data not shown), major factors for consumer’s choice. As far as nutritional compounds, we observed a small reduction in the content of sugars (**Figure 6C**), which represent the main metabolites, making up over 60% of the dry matter (Davies and Hobson, 1981), and which can affect both the taste and flavor of tomatoes. Iodine may have interfered with the metabolism of the primary compounds within the fruit (Ho, 1996) and this would be worth further evaluation.

Another important qualitative trait of tomato fruits is represented by their antioxidant power. Several studies have established a link between the dietary consumption of tomatoes, representing a major source of antioxidants, and reduced risk and prevention of important pathologies (Agarwal and Rao, 2000). Interestingly, the antioxidant capacity of tomato fruits was not influenced by the accumulation of low iodine amounts, i.e., those most appropriate for a biofortification program (**Figure 6D**). However, the fruits accumulating higher quantities of the element showed a significant increase in their antioxidant capacity (**Figures 6A,D**), thus suggesting that iodine over a certain threshold could trigger a moderate antioxidant response in the fruit, probably against the mild stress caused by the iodine itself. This is in line with similar effects detected, for example, in lettuce (Blasco et al., 2008,^2011^).

Fruit quality is a complex mixture of different traits, related, among others, to color, homogeneity, taste, flavor, size, shape, and content of nutritional compounds (sugars, acids, antioxidants). Although our results did not show major effects of iodine on the quality of the biofortified tomatoes, we analyzed only a few aspects of it. Therefore, further analyses can be performed to go into details and also to characterize other qualitative traits of the fruits.

In conclusion, we believe that the results of our study highlight several positive aspects in using tomato plants as a target for iodine biofortification programs. Plants can efficiently take up and translocate sufficient amounts of this element to the fruits, even if fertilized with low non-toxic doses of both KI and KIO_3_. On the whole, it does not seem that these processes are significantly influenced by the organic matter content of the soil or by the level of nitrate used in the fertilization of the plants, two possible factors worth considering when setting up an agronomic protocol. Of the two different iodine forms tested, KIO_3_ is preferable in order to avoid the possible, though limited, phytotoxicity problems observed in KI-treated plants. However, in soils rich in organic matter it is likely that KI maintains a higher mobility and availability for the plants. Finally, iodine-biofortified fruits appear to be suitable both for fresh market and for processing, especially if the peel is not removed.

The real efficacy of a biofortification strategy requires the careful evaluation of a series of factors. An effective and significant iodine accumulation in the edible parts of the biofortified plant and the maintenance of sufficient iodine levels when the crop is consumed, as demonstrated in this study, represent only the starting point. In fact, only biofortification protocols combining an effective micronutrient increase with high crop yields (or at least an absence of yield reductions) can be successfully adopted by a significant number of farmers. These productive aspects have been only partially tackled in the present study and certainly require a more extensive evaluation, for example in open field conditions. Finally, a tangible improvement should be demonstrated in the iodine status of those that consume biofortified tomatoes. This means that the iodine accumulated must be sufficiently bioavailable to significantly improve the original malnourished status of the consumer. An iodine bioavailability clinical trial is thus necessary as along with an analysis of the possible effects of iodine intake through tomatoes on thyroid functions.

## Conflict of Interest Statement

This research work was financially supported by SQM Europe N.V.
